# Tourism cloud management system: the impact of smart tourism

**DOI:** 10.1186/s13677-022-00316-3

**Published:** 2022-09-05

**Authors:** Fang Yin, Xiong Yin, Jincheng Zhou, Xinli Zhang, Ruihua Zhang, Ebuka Ibeke, Marvellous GodsPraise Iwendi, Mohammad Shah

**Affiliations:** 1 Business School, Sichuan University, Chengdu, 610065 China; 2 Business School, Geely University of China, Chengdu, 641400 China; 3 School of Computer and Information and Key Laboratory of Complex Systems and Intelligent Optimization of Guizhou, Qiannan Normal University for Nationalities, Duyun, Guizhou, 558000 China; 4 Faculty of Education, Language and Psychology Postgraduate Department, Segi University, Petaling Jaya, Malaysia; 5 School of Creative and Cultural Business, Robert Gordon University, Aberdeen, AB10 7AQ UK; 6 University of Nigeria, Nsukka, Nigeria; 7 University of Westminster London, London, UK

**Keywords:** Smart Tourism, Tourism, Tourists, Tourist Displacement, Data Visualization

## Abstract

This study investigates the possibility of supporting tourists in a foreign land intelligently by using the Tourism Cloud Management System (TCMS) to enhance and better their tourism experience. Some technologies allow tourists to highlight popular tourist routes and circuits through the visualisation of data and sensor clustering approaches. With this, a tourist can access the shared data on a specific location to know the sites of famous local attractions, how other tourists feel about them, and how to participate in local festivities through a smart tourism model. This study surveyed the potential of smart tourism among tourists and how such technologies have developed over time while proposing a TCMS. Its goals were to make physical/paper tickets redundant via the introduction of a mobile app with eTickets that can be validated using camera and QR code technologies and to enhance the transport network using Bluetooth and GPS for real-time identification of tourists’ presence. The results show that a significant number of participants engage in tourist travels, hence the need for smart tourism and tourist management. It was concluded that smart tourism is very appealing to tourists and can improve the appeal of the destination if smart solutions are implemented. This study gives a first-hand review of the preference of tourists and the potential of smart tourism.

## Introduction

Destinations like China are primed for successful performance in incoming tourism, with China set to overtake France as the leading destination worldwide by 2030 [1]. Similarly, tourism in the city of Mumbai, India, also increases yearly. Between 2009 and 2017, Mumbai saw an increase of 10.6%— the second highest growth rate in tourism for European tourists. The World Travel Awards awarded India and Mumbai the ‘Best Destination’ and ‘Best Destination City’, respectively, in 2018. However, China is primed to take over this position. As tourism steadily grows, so does the use of smartphones. As observed in [2], the cellphone has been adopted as a regular tourist travel device because of its ubiquity capabilities and progressive computing. With exponential growth in its users and its growing variety of applications, the mobile phone is changing the perspective of tourist travel and transport networks. The capacity to carry over 8 sensors with context-awareness opportunities is what has made smartphones so valuable in tourism [3]. The camera and microphone, the first features of the smartphone, have quickly been joined by GPS, compasses, proximity sensors, accelerometers, and gyroscopes. These proximity sensors allow system-adaptive devices to develop and function effectively as smart personal assistants. The two examples below could make the experience easier:

- A smartphone with a mobile app which uses the camera and QR code technology for the validation of tickets, making the use of paper tickets redundant.
- The use of Bluetooth and GPS for real-time identification of tourists’ presence, following dynamic patterns and enhancing the transport network.

The main contribution of this paper is the analysis of methodologies that include the features mentioned above in already existing technologies while proposing a Tourism Cloud Management System (TCMS). We consider a mobile which could be used to sell reserves for the transport of tourists, using a QR code as an identifier for validation. We also consider a mobile app which gives tourists access to the digital form of their travel reservations and makes it easy for them to find stops for transport [4].

Following the growth and development of the tourism industry, innovations in finding customers, linking them, and assuring their satisfaction need to be enhanced. The competition for prices, especially for holiday destinations, is high. Information Technology allows for greater efficiency, swift reaction time, and reduced operational costs. Recent advancements have inspired extreme changes in the tourism industry. A useful development in tourism is the redesign of the booking interaction, which would allow shoppers to save time in the acquisition and reservation of tourist items. Thus, tourism organizations need to adopt cutting-edge innovations to enhance their all-around productivity.

### Literature review

**Fig. 1:**
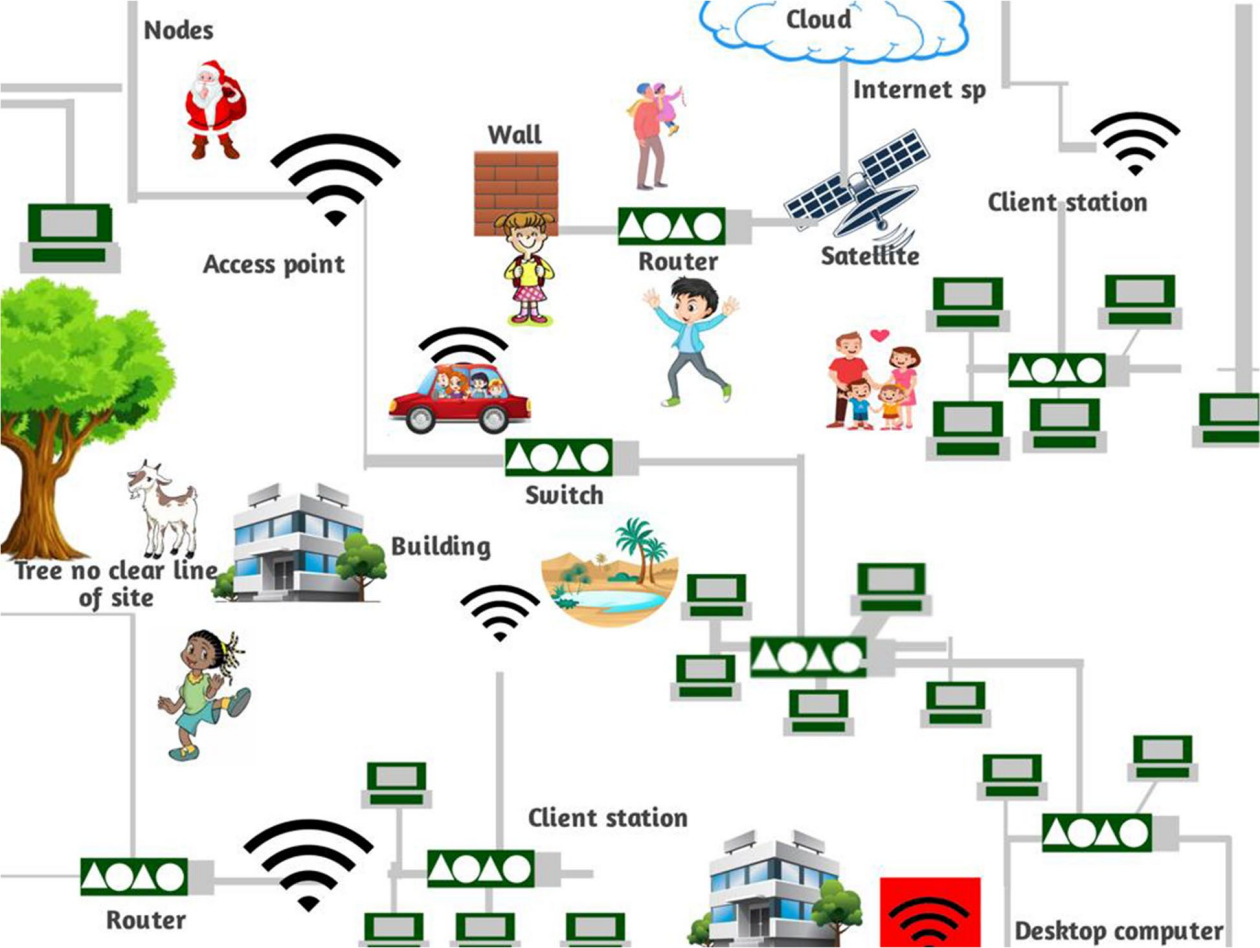
Tourism Cloud Management System (TCMS) Architecture

Information and Communication Technology (ICT) has given people access to infinite digital content on leisure, culture, education, etc., on their portable devices [5]. The architecture of the Tourism Cloud Management System (TCMS) is shown in Fig. 1. The evolution of technology has changed consumer behavior in various fields, and the tourism industry isn't an exception. ICT brings several advantages, including automation of processes which increases speed, reduced errors, and lowered expenses, all of which are of prime importance in the tourism industry. Secure processes which allow the purchase and marketing of products and services, and the transfer of videos, images, etc., are things which generate motivation for leisure-seeking consumers [6]. The tourist market is dynamic in terms of demand trends of tourists, which varies consumption habits and allows the generation of various products. This led to the creation of a route model called the intelligent tourist route to address this need.

The concept of smart territory in recent years has become popular among researchers and scholars of sustainable development. From the point of view of architecture and urbanism, the authors in [7] came up with the concept of smart territory and defined it as innovative territories with the ability to build competitive advantages for their surroundings, in the framework of a complicated and interconnected world. These smart territories try to find a balance between sustainability, social cohesion, and economic competition. Any territory, notwithstanding its level of infrastructure or size, can be a smart territory.

The definition of smart territory evokes a novel concept of the confluence of various related disciplines including culture, heritage, architecture, environment, regional development, urban planning, and the economy of innovation. The multidisciplinary approach of distinct sciences is applied and generates a synergy to consolidate a framework for tourism to be used in smart tourist destinations [8].

The multidisciplinary capacity and the integration of various areas need to be focused on the development of infrastructure for sensitization and connectivity. The concept of smart territory is especially relevant when one considers that the field of social and economic development needs to be approached from different perspectives. This means that the existing approach to the economy needs to have a holistic system with attractions, facilities with local management, quality and affordable pricing. This would allow the concept of a product in a destination, where circuits and routes form part of the activities.

### Data visualization as a consolidation tool for smart routes

An essential part of the work done in tourism is the data visualization from the inventory of tourist attractions. It is important because it allows for a practical alternative for the conceptualization of the activity from the view of technological innovation and development in the conventional approach to tourism [9]. A good approach to the generation and consolidation of tourism science is the integration of different disciplines, with the view of creating synergy. This is exemplified by the research of evaluation and registration of tourist heritage elements (PT), connected to the data visualization (open data) created by computer graphics. Computer graphics allow the use of the most advanced technology; sensory interpretation through the perception, modelling, and representation of objects in 2D and 3D animation. These computer apps are not applied in tourist activities.

Visualization of data is a concept which uses the large correspondence force of pictures to clarify the cause, reliance, and importance found in the conceptual masses of data produced by social and logical cycles. It has not yet been fully explored in the field of tourism. It is a technological experiment in tourism management and planning as a result of its structure and the order of cultural and natural attractions. They are usually applied with the view that they can determine their possible uses [10]. An important part of the process of analyzing the tourist potential of a territory is the categorization, ranking, and inventory of tourist attractions. These processes determine how the real tourist vocation of territories would be. Based on its scope of application, it is the basis for organizing the tourist space of a community, region, department, country, or municipality. Thus, it is essential to consider tourist inventory for the generation of circuits and routes.

The only way to verify the tourist potential of the territory and justify possible investments is through a strict examination of the demand, supply, market trends, competition, and attractions of the territory. All these factors make up a comprehensive assessment of the territory and a basis for development agencies to make their decisions.

The valuation of the tourist territory consists of three primary stages:

1. Evaluation of the existing supply, resources, demand, market trends, and competition in the territory.
2. Comparison of the results of the evaluation and identifying the strengths, weaknesses, risks, and opportunities of the territory.
3. If there is potential, a strategy needs to be defined and followed for the development of tourism in the territory [11].

These three stages consist of the collation, treatment, and evaluation of external and internal information about the territory with strong tourism potential for the Tourism Cloud Management System.

### Implementation of augmented reality and TCMS

The internet has caused a revolution in companies, tourism, the population, and the world in general. In the tourism space, it has facilitated a change in the sales and consumer channel, which reduces third-party participation and enhances competition. Consumers who are connected to the digital world with smartphones and can decide what they want have also revolutionized the world. Agents in the tourism industry have had to adapt to novel innovative technologies and the reformation of their customary products. These new technological advances make the products more valuable, allowing for a better experience [12]. Success in the management of a tourist destination can only be done with the detection of changes in the environment and their effects [13]. Adapting to new technologies is important because of the ease of promoting a destination.

TCMS shows that tourists in search of pleasurable experiences employ technological devices to speed up the selection process of what they are looking for. The perception of textual and graphical information of mobile disposition is very essential for this, as well as the possibility of reading other people’s opinions from their reviews. The options for accessing this information are numerous, including augmented reality which is on the rise [14]. For instance, a foreign tourist, a single male of higher education and 36 years of age would use the internet for planning his trip because he has spent most of his life in the technological era. It is important to know your tourist destination before getting there.

Augmented reality is an innovative tool that integrates the virtual world with the real one. It displays the content in real-time through a gadget. The difference between virtual reality and augmented reality is that the former displays elements virtually, while the latter displays real elements in a space. Although this technology has been in use since the 90 s, it is only recently adapted to mobile devices. The incorporation of this technology into tourism allows for the massive promotion of destinations and includes media information which complements the visit of the tourists in real-time. The application of augmented reality in tourist routes would allow easy access to information that can be obtained from the web. There are infinite possibilities for obtaining tourist information. This is a step towards an intelligent destination [15] in line with other state-of-the-art procedures in big data analysis [16–20].

### Application of the smart tourist destination management model

The concept of a smart tourist destination should not be considered only as the application of the smart city model to the tourism industry. Structural variations in the tourism industry, which integrate with the consolidation of the smart city model, form the basis of the need for novel approaches to managing tourist destinations [21]. Thus, it is important to analyze some definitions of the concept. The India Tourism Ministry defined Smart Tourist Destination (STD) as an accessible innovative space, integrated with state-of-the-art technology that ensures the territory’s sustainable development, accelerates the interaction of the visitor and the environment, and enhances the quality of their trip. The basis of this definition is the principle that innovation is an essential space and operates as the centre of all proposals. According to [3], it inaugurates innovative spaces such as STDs that use novel technologies for development.

This methodology combines the novel framework of the tourist region with the climate and communication of the guests to expand the nature of their experience. It uses ICT to improve administration, reduce costs, and enhance residents’ satisfaction. The White Book on STDs emphasizes the development and innovation of ICT as the basis for novel mechanisms for the promotion of STDs. The goal is to create various differential competitive services with profitability and sustainability that turns risk into opportunity, promote diversification, fights against seasonality, and integrates success.

Gretzel et al. [2] defined STD as novel models of business management, forms of communication, and the quest for the consumer’s well-being. They use ICT for the promotion of the sustainable development of the territory, efficient management of resources, facilitation of interaction between the environment and visitors, and enhancing the citizens’ quality of life [3]. An STD’s configuration must correspond to the needs of every destination and their benefits because the mere application of technology doesn’t automatically make a destination an STD. It must be followed by a series of changes at all levels. An STD must use the available tools to satisfy the market, and because technology is not a means to an end, issues regarding accessibility, innovation, and sustainability must still be considered. Authors in [3] considered the concept of STD as a generic architecture that consists of principles originating from smart cities and includes sustainability and competitiveness at its base. The aim is to provide a holistic structure for smart tourist destinations. The idea is to revolutionize the management of tourism according to local capacity and technological possibilities.

## Summary of the related recent works

**Table:** 

S/N	Author(s)	Description
[5]	Gretzel, U., Werthner, H., Koo, C. and Lamsfus, C	With the aid of smart business networks and digital ecosystems, this paper illustrates the concept of a smart tourism ecosystem
[6]	Buhalis, D. and Amaranggana, A	This paper contributes knowledge as to how smart tourism destinations can improve the tourism experience by offering personalized products and services to users
[7]	Boes, K., Buhalis, D. and Inversini, A	This paper examines the dimensions for which technology is vital for the development of smart tourist destinations and smart cities
[8]	Hunter, W.C., Chung, N., Gretzel, U. and Koo, C	This paper proposes the constructivist framework and related research methodologies for understanding how smart tourism is a type of soft power
[9]	Tribe, J. and Mkono, M	This paper conceptualizes the concept of e-lienation as a kind of alienation in ICT-based tourism
[10]	Gretzel, U., Zhong, L. and Koo, C	This paper explores the concept of smart tourism cities and the role urban cities have to play in it
[11]	Jovicic, D.Z	This paper reviews how tourism destination concepts have evolved, to examine how the term ‘destination’ has changed over the years
[12]	Shafiee, S., Ghatari, A.R., Hasanzadeh, A. and Jahanyan, S	This paper presented a novel model for smart tourism destinations with the aid of the grounded theory as an analytical architecture
[13]	Koo, C., Shin, S., Kim, K., Kim, C. and Chung, N.,	This paper focuses on the effectiveness of smart tourism in Korea, and how it delivers travel information
[14]	Del Chiappa, G. and Baggio, R.,	This paper applies an analytic approach to the case study of three tourism destinations to understand the processes and transfer of information
[15]	Boes, K., Buhalis, D. and Inversini	This paper studies the concepts of ICT, social and human capital as important components of smart tourism destinations
[16]	Ngabo, D., Dong, W., Ibeke, E., Iwendi, C. and Masabo, E	This paper introduces an AI algorithm to predict the rate of survival of COVID-19 based on the patient’s features
[17]	Palanivinayagam, A., Gopal, S.S., Bhattacharya, S., Anumbe, N., Ibeke, E. and Biamba, C	This paper had the objective of removing prime attributes to improve the accuracy of the subject machine learning algorithm to reduce crime
[18]	Iwendi, C., Maddikunta, P.K.R., Gadekallu, T.R., Lakshmanna, K., Bashir, A.K. and Piran, M.J	This paper aims to reduce the energy consumed by IoT sensors that may improve the network’s lifetime
[19]	Iwendi, C., and Allen, A.R	This paper proposes a network with an efficient and dynamic key distribution scheme to prevent algorithm complications and attacks
[20]	Ch, A., Ch, R., Gadamsetty, S., Iwendi, C., Gadekallu, T.R. and Dhaou, I.B	This paper proposes a model to predict natural disasters and identify drought and flood conditions
[21]	Del Vecchio, P., Mele, G., Ndou, V. and Secundo, G	This paper illustrates how the large amount of social big data obtained from tourists can develop the value creation process for a smart tourism destination

### Analysis of smart tourism using scopus

**Fig. 2:**
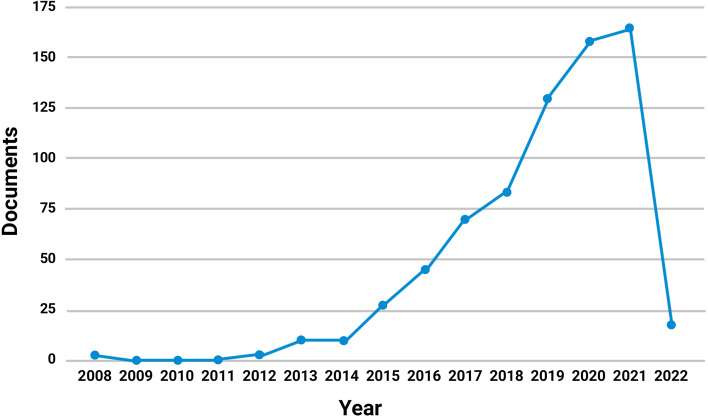
Number of documents related to ‘smart tourism’ from 2008 to 2022 in the SCOPUS database

The term ‘smart tourism’ was searched in the keywords, abstract, and titles of the extant SCOPUS literature. Between 2008 and 2022, 710 documents were published. These documents were studied for analysis. Figure 2 shows the published papers related to ‘smart tourism’ from 2008 to January 2022.

**Fig. 3:**
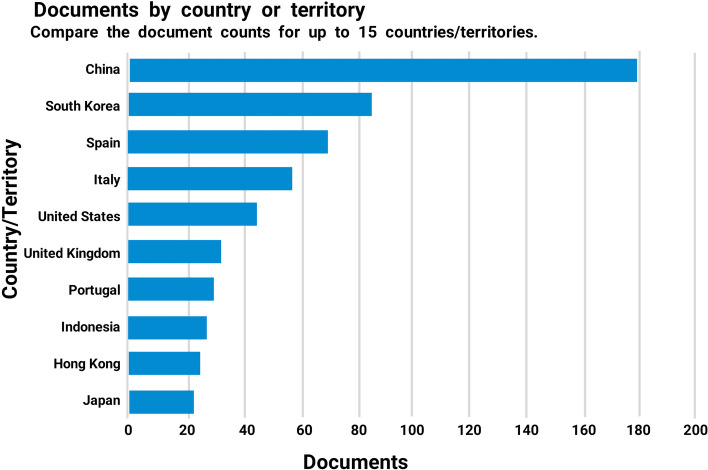
Documents by country related to ‘smart tourism’

Figure 3 illustrates the top 15 countries that have published work linked to smart tourism from 2008 to 2022.

**Fig. 4:**
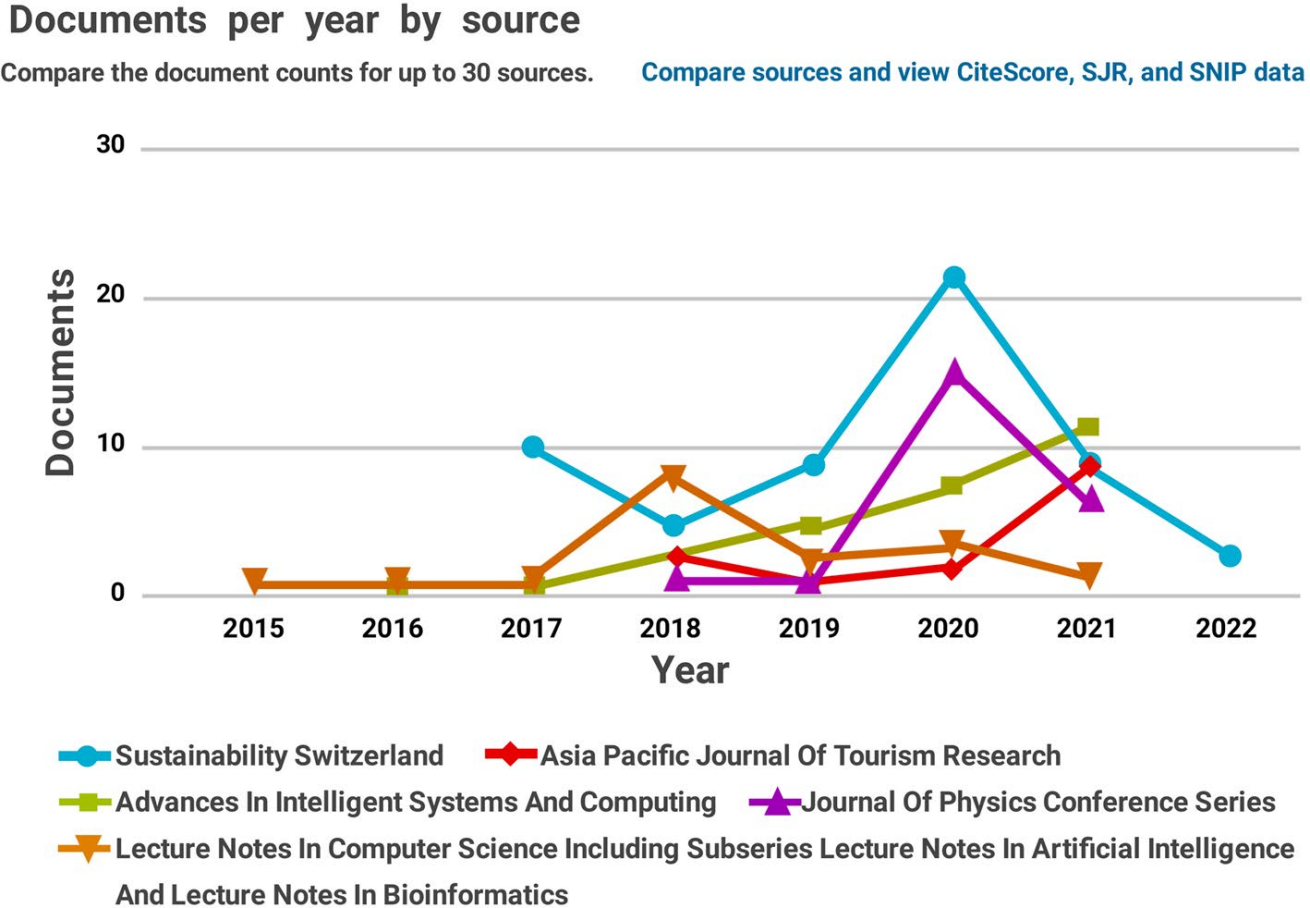
Documents per year by source related to ‘smart tourism’

Figure 4 illustrates the comparison of published papers on smart tourism per year.

The data collated from SCOPUS was further examined with the VOS viewer. Table 1 shows the list of countries with 5 or more publications on smart tourism.

**Table 1:** Citations by country

SR. NO	COUNTRY	DOCUMENTS	CITATIONS	TOTAL LINK STRENGTH
1	SOUTH KOREA	82	2613	1049
2	UNITED STATES	46	2331	942
3	CHINA	179	1704	712
4	AUSTRALIA	14	1328	599
5	SPAIN	71	1203	906
6	ITALY	58	852	466
7	UNITED KINGDOM	32	727	370
8	SWITZERLAND	7	561	43
9	AUSTRIA	12	437	330
10	TAIWAN	20	409	219
11	UNITED ARAB EMIRATES	10	366	173
12	HONG KONG	23	310	341
13	MALAYSIA	14	256	79
14	PORTUGAL	28	221	217
15	TURKEY	10	203	55
16	MACAU	13	195	191
17	CANADA	8	191	39
18	INDIA	17	174	50
19	GERMANY	12	166	140
20	SERBIA	6	160	94
21	BRAZIL	16	125	218
22	IRAN	9	87	135
23	CROATIA	5	83	62
24	RUSSIAN FEDERATION	17	81	81
25	JAPAN	22	74	108
26	COLOMBIA	7	69	43
27	MEXICO	6	45	18
28	INDONESIA	27	41	134
29	VIET NAM	5	31	12
30	POLAND	8	28	78
31	GREECE	9	25	42
32	SLOVAKIA	5	24	58
33	ROMANIA	5	10	16
34	MOROCCO	6	9	11
35	THAILAND	5	3	21
36	ECUADOR	5	0	28

**Fig. 5:**
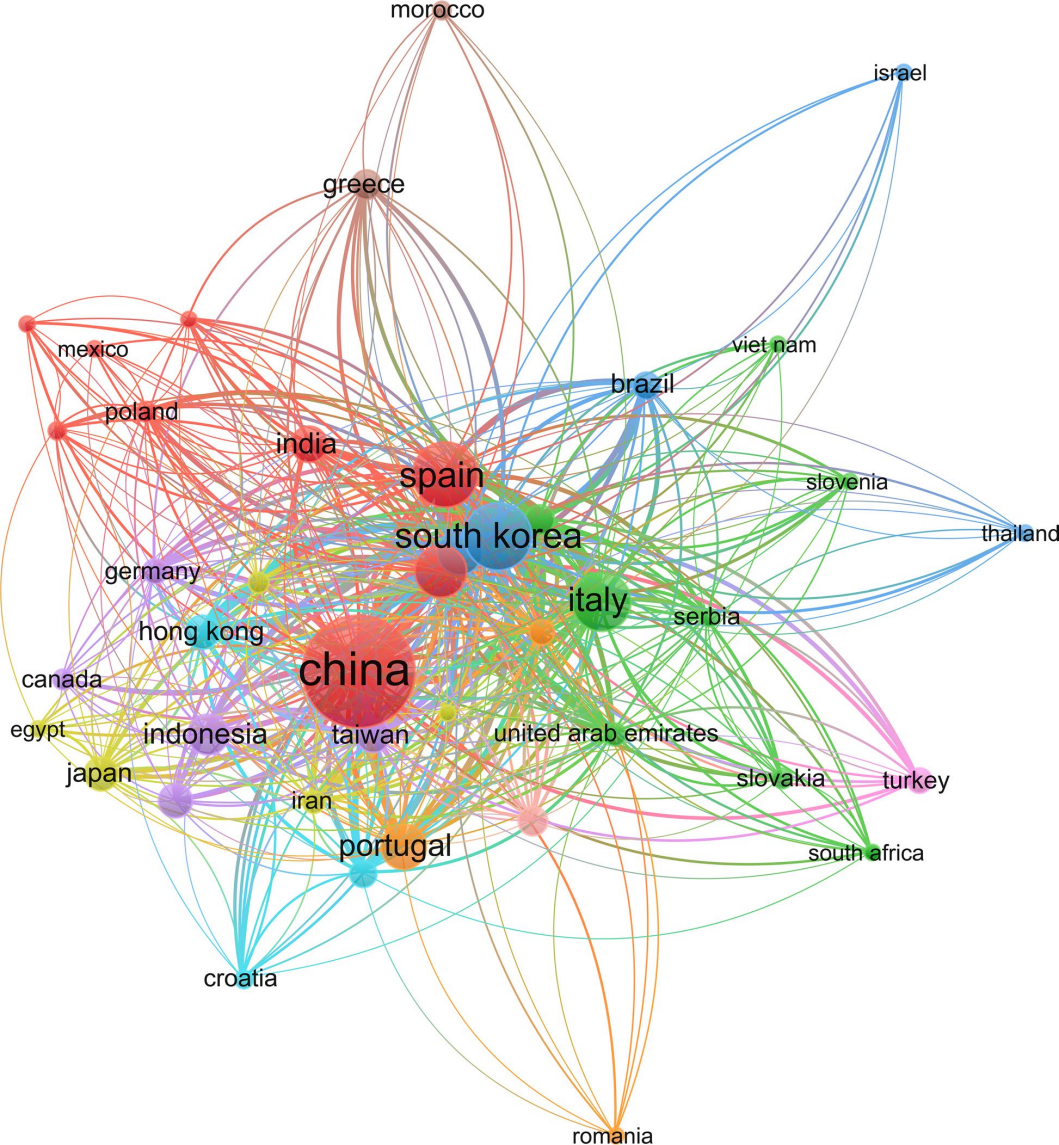
Citations by country

Figure 5 visualizes the citations by country in Table 1.

**Fig. 6:**
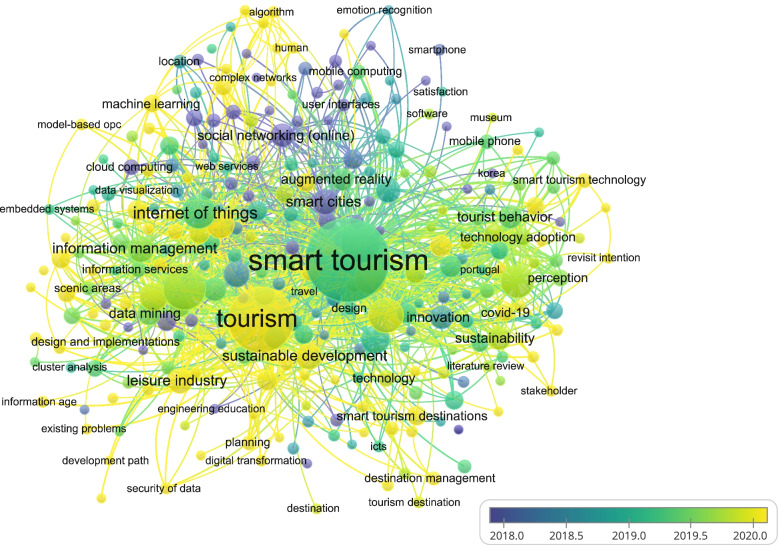
Keywords: Overlay visualization

Figure 6 illustrates the keywords used in the published works of smart tourism, including the recent research of 2020. Some of these keywords include smart tourism, city, climate change, e-tourism [22], etc.

## Methodology

The TCMS study in this research uses a descriptive approach: a survey with a questionnaire to collect the required data. We formed a respondent base of 200 tourists in China selected based on the reviews of popular tourist portals including Trip Advisor. All tourists were given a link to a Google form (questionnaire) they were to fill out. 66 completed responses were used for the analysis. The questionnaire comprised close-ended questions using Likert’s five-point scale. The collated data were evaluated statistically with SPSS (Statistical Package for the Social Sciences), a statistical software used for advanced analytics, data management, business intelligence and multivariate analysis.

## Results

Table 2 shows the percentage of people who admitted they take trips to unknown destinations very often.

**Table 2:** How often do you travel to unknown places in a year?

	***Frequency***	***Percent***	***Cumulative Percent***
**Not so often**	10	15.2	15.2
**Quite often**	2	3.0	18.2
**Very often**	54	81.8	100.0
**Total**	66	100.0	

Table 3 shows the percentage of consumers who believed that tourist interests would increase and the percentage of consumers who believed the level of interest would remain static.

**Table 3:** Will the implementation of smart solutions in tourist places generate interest among the tourists?

	***Frequency***	***Percent***	***Cumulative Percent***
**Decrease**	4	6.1	6.1
**Definitely Increase**	20	30.3	36.4
**Might Increase**	16	24.2	60.6
**Stay Same**	26	39.4	100.0
**Total**	66	100.0	

Table 4 illustrates the percentage of respondents who said they would be satisfied with the quality of smart tourism application services.

**Table 4:** Will smart tourism applications enhance the quality of service?

	***Frequency***	***Percent***	***Cumulative Percent***
**Neutral**	20	30.3	30.3
**Not Satisfied**	14	21.2	51.5
**Satisfied**	22	33.3	84.8
**Very Satisfied**	6	9.1	93.9
**Very Unsatisfied**	4	6.1	100.0
**Total**	66	100.0	

Table 5 shows the percentage of respondents who believe they would communicate better in a foreign land with smart tourism applications.

**Table 5:** Will smart tourism applications result in better communication in foreign lands?

	***Frequency***	***Percent***	***Cumulative Percent***
**Definitely Yes**	18	27.3	27.3
**No**	28	42.4	69.7
**Yes**	20	30.3	100.0
**Total**	66	100.0	

Table 6 shows the mean for males and females and the mean difference at the threshold p-value of lower than 0.05 level. The F-test value was 0.536, indicating it was significant at 0.465, and that the variance of both groups is equal. The equal variance was used as per the T-test that was conducted. The T value for the equal variances was 2.774, meaning it was significant at 0.006, which indicates a difference in male and female behavior regarding the preference for smart tourism.

**Table 6:** Analysis of T-test

	Levene’s Test for Equality of Variances		t-test for Equality of Means						
	**F**	**Sig**	**T**	**df**	**Sig (2-tailed)**	**Mean Diff**	**Std Error Diff**	**95% Confidence Interval of the Difference**	
								**Lower**	**Upper**
Equal Variance Assumed	.536	.465	2.774	385	.006	4.148	1.496	1.208	7.090
Equal Variance Not Assumed			2.718	226.62	.007	4.148	1.526	1.141	7.156

Many people go to tourist destinations annually and believe that their interest may increase if smart solutions are available. The majority agreed that the quality of service may be enhanced if there are smart tourism applications. However, most did not believe that smart tourism applications may result in better communication in foreign lands. From the results presented above, it is obvious that the implementation of smart tourism applications would result in better patronage from tourists for tourist destinations. The results illustrate that in comparison with state-of-the-art existing work, there are several possibilities and applications of it in multiple fields.

## Conclusion

This paper proposes a Tourism Cloud Management System (TCMS) and discusses the infinite possibilities of the comprehensive approach that integrates different disciplines for the consolidation of a multi-and transdisciplinary concept based on limited action and functionality. It includes several possibilities in design, tourism, ICT, economy, and multimedia, among others. The implementation and application of the TCMS would enable intelligent and smart management of tourism and enhance tourists' experiences. It is necessary to take local action to integrate the proposed Smart Tourist Destination Management model. In the future, this paper aims to systemize and homogenize the collection of information on various tourist attractions, i.e., making the collection of information on tourist attractions easier. Another objective is to propose its use in preparing sustainable development plans for tourism and to convince private and public individuals to support the use of technological innovation in tourism.

## Data Availability

The supporting data can be provided on request.
